# Prognostic Significance of Tumor-Infiltrating Natural Killer Cells in Solid Tumors: A Systematic Review and Meta-Analysis

**DOI:** 10.3389/fimmu.2020.01242

**Published:** 2020-07-02

**Authors:** Shuo Zhang, Weijian Liu, Binwu Hu, Peng Wang, Xiao Lv, Songfeng Chen, Zengwu Shao

**Affiliations:** ^1^Department of Orthopaedics, Union Hospital, Tongji Medical College, Huazhong University of Science and Technology, Wuhan, China; ^2^Department of Orthopaedic Surgery, The First Affiliated Hospital of Zhengzhou University, Zhengzhou, China

**Keywords:** tumor-infiltrating NK cells, NK cell markers, solid tumor, prognosis, meta-analysis

## Abstract

**Background:** Tumor-infiltrating natural killer (NK) cells (TINKs) are crucial immune cells in tumor defense, and might be related to tumor prognosis. However, the results were discrepant among different studies. The present meta-analysis was performed to comprehensively assess the prognostic value of NK cell markers in solid tumor tissues.

**Methods:** PubMed, Web of Science, and EMBASE were searched to identify original researches reporting the prognostic significance of TINKs in solid tumors. NK cell markers CD56, CD57, NKp30, and NKp46 were included in the analysis. The hazard ratios (HRs) and 95% confidence intervals (CIs) of pooled overall survival (OS), disease-free survival (DFS), metastasis-free survival (MFS), progression-free survival (PFS), and recurrence-free survival (RFS) were calculated by STATA software 14.0 to assess the prognostic significance.

**Results :** Of the 56 included studies, there were 18 studies on CD56, 31 studies on CD57, 1 study on NKp30, and 7 studies on NKp46. High levels of CD56, CD57, NKp30, and NKp46 were significantly correlated with better OS of patients with solid malignancies (HR = 0.473, 95%CI: 0.315–0.710, *p* < 0.001; HR = 0.484, 95%CI: 0.380–0.616, *p* < 0.001; HR = 0.34, 95%CI: 0.14–0.80, *p* = 0.014; HR = 0.622, 95%CI: 0.470–0.821, *p* < 0.001, respectively). Our results also revealed that CD56, CD57, and NKp46 could act as independent prognostic predictors for favorable OS (HR = 0.372, 95%CI: 0.261–0.531, *p* < 0.001; HR = 0.525, 95%CI: 0.346–0.797, *p* = 0.003; HR = 0.559, 95%CI: 0.385–0.812, *p* = 0.002, respectively).

**Conclusions :** Our results indicated that high levels of NK cell markers in solid tumor tissues could predict favorable prognosis for solid tumor patients.

## Introduction

With a high morbidity and mortality rate, malignant tumor is regarded as a significant health problem around the word, which results in heavy medical and socioeconomic burdens (1, 2). Despite substantial advances in the diagnosis and therapeutics of malignancies in recent decades, poor tumor prognosis is still an issue for researchers and clinicians (3). Therefore, more and better biomarkers are urgently needed to facilitate tumor diagnosis, predict tumor prognosis, and develop novel therapies (4).

Tumors develop and progress in a microenvironment that contains various immune cells and immune products (5). Generally, natural killer (NK) cells are considered to constitute the first line of anti-tumor defense, and provide tumor immunosurveillance, tumor lysis, and elimination of tumor metastasis (6). NK cells can surveil tumor progression by recognizing the lacking expression of major histocompatibility complex (MHC) class I molecules in tumor cells (7). A higher incidence of tumor was observed in the population with low NK cell cytotoxic activity, which further suggested that NK cells played a vital role in tumor immunosurveillance (8, 9). According to the classical NK cell activation model, NK cells directly destroy tumor cells in an MHC-unrestricted manner without the help of antigen-presenting cells (10, 11). Perforin, granzyme, and factor associated suicide ligand (FasL) pathway are all involved in NK cell-mediated tumor cell lysis (12, 13). Additionally, NK cells can release multiple soluble anti-tumor factors, such as interferon (IFN)-γ and tumor necrosis factor (TNF)-α (10, 11).

NK cells can acquire altered phenotypes and function states in the tumor microenvironment due to their plasticity (14). Although a number of tumor-infiltrating NK cells (TINKs) were detected in solid tumors, increasing evidence revealed that tumor cells might evade the immunosurveillance of NK cells. Tumor cells could downregulate the expression of activating surface receptors in NK cells, such as natural killer group 2 member d (NKG2D) (15, 16). In addition, tumor cells could not only decrease the expression of IFN-γ, TNF-α, and interleukin (IL)-2 by NK cells, but also transfer TINKs and peripheral blood (PB) NK cells (PBNKs) to an anergic or low-cytotoxic state (17). Sun et al. demonstrated that the expression of CD96 was associated with the functional exhaustion of NK cells in hepatocellular carcinoma (HCC) (18). Moreover, previous studies also showed the differentiation potentials of TINKs to a protumorigenic or proangiogenic phenotype (19, 20). Taken together, NK cells exert bidirectional effects in tumorigenesis and the explicit role of TINKs in malignancies needs to be further studied.

Accumulating evidence suggests that NK cells are composed of multiple sub-populations with different phenotypes and function states (21–23). A variety of surface markers have been used to label different NK cell subsets and to measure their functional properties (Table 1). In several clinical observation studies, the degree of NK cell tumor-infiltration was found to be highly associated with the prognosis of solid tumors (27, 28). However, the clinical significance of NK cells in tumor defense seems to be controversial due to previously reported discrepant results (29, 30). Therefore, the current meta-analysis was performed to comprehensively assess the prognostic value of the NK cell markers in all types of solid tumors.

**Table 1:** Surface markers of NK cells.

**Surface marker**	**Function**
CD56 (NCAM1)	CD56 could drive the maturation of NK cells. CD56^bright^ NK cells are weak in cytotoxicity, but strong in the production of anti-tumor cytokines such as IFN-γ and TNF-α (24).
CD57 (HNK-1, LEU-7)	CD57 is regarded as a marker of terminal differentiation. CD57^+^ NK cells are less proliferative but more cytotoxic to tumor cells compared with CD57^−^ NK cells (25).
NKp46 (NCR1, CD335)	Activating receptor.
NKp44 (NCR2, CD336)	Activating receptor NKp44 is expressed in activated NK cells, but not in resting NK cells. MLL5 is the ligand of NKp44 (26).
NKp30 (NCR3, CD337)	Activating receptor. BAG6 and NCR3LG1 are the ligands of NKp30 (26).

*NCAM1, neural cell adhesion molecule 1; HNK-1, human natural killer-1; NCR, natural cytotoxicity receptor; MLL5, mixed-lineage leukemia; BAG6, BCL2-associated athanogene 6; NCR3LG1, NCR3 ligand 1*.

## Methods

### Search Strategy

Two investigators (SZ and WL) independently conducted the systematic literature retrieval in PubMed, Web of Science, and EMBASE to obtain eligible researches up to October 1, 2019. The search query was “natural killer cell OR NK cell OR CD57 OR CD56 OR NKp30 OR NKp46 OR NCR OR natural cytotoxicity receptor” AND “cancer OR tumor” AND “survival OR prognosis.” Moreover, the references of relevant reviews or articles were screened for eligible studies.

### Study Selection

Original research was included if it satisfied all the following criteria: (1) Studies examined the levels of NK cell markers (CD56, CD57, NKp30, and NKp46) in solid tumor tissues of patients; (2) The tumor cases were divided into two groups according to the levels of NK cell markers; (3) Studies explored the association between the levels of NK cell markers and overall survival (OS), disease-free survival (DFS), metastasis-free survival (MFS), progression-free survival (PFS), or recurrence-free survival (RFS) in solid malignancies; and (4) Sufficient data were reported in the publications for us to obtain hazard ratios (HRs) and 95% confidence intervals (CIs).

Exclusion criteria included: (1) Studies on the association between tumor prognosis and PBNKs, (2) Studies not in English, (3) Studies not on humans, and (4) Repetitive studies. If different publications reported duplicate data, only the latest one was included.

### Data Extraction and Quality Assessment

Two researchers (SZ and XL) reviewed the included articles carefully and extracted the following study characteristics: author's name, year of publication, region, tumor type, clinical stage, sample size, endpoint, detection method of NK cell markers, and follow-up period. HRs and 95% CIs served as reasonable parameters in the data analysis. If the HRs and 95% CIs could not be directly obtained from the publications, the data were extracted from the Kaplan-Meier curves using Engauge Digitizer version 9.8.

The quality of the included studies were evaluated according to the Newcastle-Ottawa Scale (NOS), especially for cohort studies. NOS, ranging from 0 to 9, includes three domains: selection of the exposed cohort, comparability of the cohorts, and assessment of the outcome. Studies with a NOS score more than 6 were recognized as high-quality studies. Rating of the study quality was conducted by two independent raters (PW and BH) and disagreements were resolved by a third researcher (SC).

### Data Analysis

HRs and 95% CIs were log-transformed and pooled by Stata statistical software version 14.0 (Stata Corporation, College Station, TX, USA). The heterogeneity among the research was examined by Higgins *I*^2^ statistic and Cochran Q test. A fixed-effect model was conducted if the heterogeneity was not significant (I^2^ < 50%); otherwise (*I*^2^ ≥ 50%), random-effect model was considered to be more suitable. The result that pooled HR was less than 1 indicated a better prognosis in the patients with a high-degree of NK cell tumor-infiltration. In addition, we evaluated the stability and credibility of summarized outcomes by sensitivity analysis and assessed the potential of publication bias by Begg's test (31). We further analyzed the prognostic significance of NK cell markers in certain types of tumors. *P*-values were two-sided, and *P* < 0.05 was considered to be statistically significant.

## Results

### Study Characteristics

**Figure 1:**
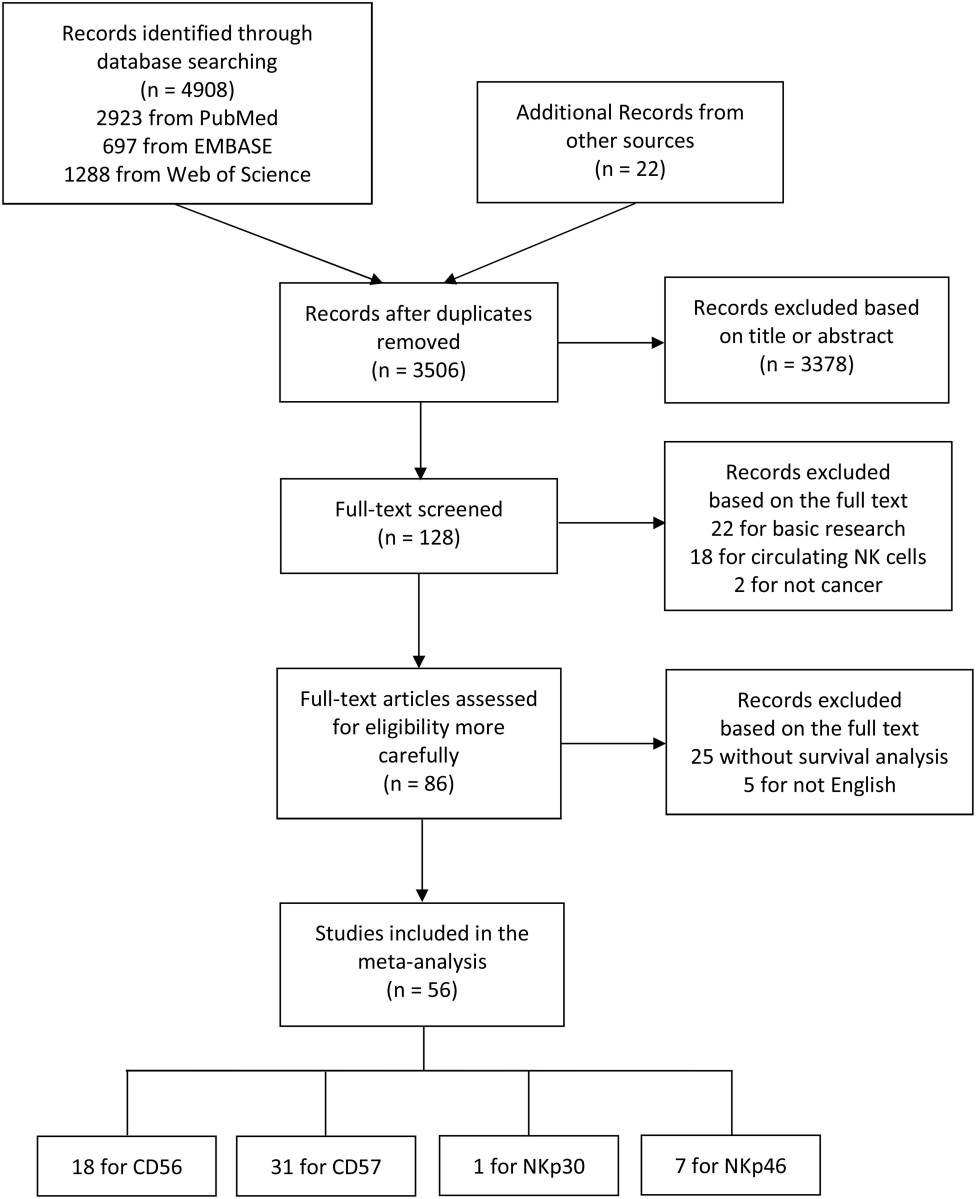
The flow diagram indicating the process of study selection.

The flow diagram of literature retrieval and selection was shown in Figure 1. Among 56 included articles, there were 18 studies comprising 20 cohorts on CD56 (27, 30, 32–47), 31 studies on CD57 (29, 35, 48–76), one study on NKp30 (77), and seven studies on NKp46 (78–84). The study characteristics and NOS scores of included researches are shown in detail in Supplementary Tables 1, 2, respectively. To summarize these in brief: (1) the sample size ranges from 16 to 1259; (2) the publication year ranges from 1997 to 2019; (3) 52 studies performed immunohistochemical (IHC) staining to evaluate NK cell markers, while one study conducted flow cytometry (FCM) analysis, and three studies conducted real-time polymerase chain reaction (RT-PCR); (4) HRs and 95%CIs were not directly obtained from eighteen included studies; and (5) the NOS score ranges from 6 to 8.

### Prognostic Value of NK Cell Marker CD56 in Patients With Solid Tumors

**Figure 2:**
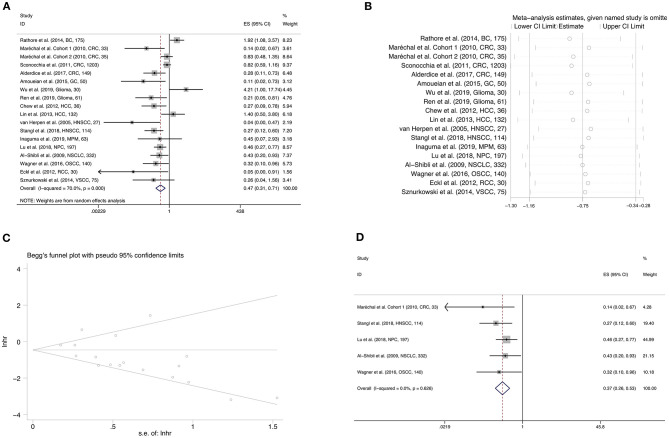
Forest plot **(A)**, sensitivity analysis plot **(B)**, and Begg's test **(C)** of the meta-analysis of OS for solid tumor patients divided by the level of CD56. Forest plot **(D)** of the meta-analysis of OS for solid tumor patients divided by the level of CD56, from Cox multivariate analysis. In the forest plots, each study ID was set as the following format: authors (year, tumor type, sample size).

Pooled results of 18 studies comprising 2,882 patients revealed that CD56 was significantly correlated with better OS (HR = 0.473, 95%CI: 0.315–0.710, *p* < 0.001) (Figure 2A; Table 2). The random-effect model was more appropriate owing to the obvious heterogeneity among the included studies (Cochrane Q, *p* < 0.001; *I*^2^ = 70.0%). In the sensitivity analysis, the elimination of any cohort failed to change the statistical significance, which further confirmed the stability and credibility of pooled results (Figure 2B). Begg's test indicated significant publication bias in the analysis of the association between CD56 and OS (*p* = 0.049) (Figure 2C). Moreover, pooled HR of 5 pieces of research which performed Cox multivariate analysis showed that a high level of CD56 served as an independent prognostic predictor for favorable OS of solid tumor patients (HR = 0.372, 95%CI: 0.261–0.531, *p* < 0.001) (Figure 2D; Table 2).

**Table 2:** Pooled HR, heterogeneity, and publication bias of the meta-analysis of OS, DFS, MFS, PFS, and RFS in patients with solid tumors.

**Factor**	**No. of cohorts**	**Cases**	**Model**	**Pooled HR (95%CI)**	***P***	**Heterogeneity**		**Publication bias (P of Begg's test)**
						***I*^2^**	***P***	
**CD56**								
OS	18	2,882	R	0.473 (0.315, 0.710)	<0.001^*^	70.0%	<0.001	0.049
OS from M	5	816	F	0.372 (0.261, 0.531)	<0.001^*^	0.0%	0.626	—
DFS	2	245	F	0.274 (0.111, 0.679)	0.005^*^	2.9%	0.310	—
MFS	1	114	—	0.27 (0.13, 0.55)	<0.001^*^	—	—	—
PFS	4	379	R	0.533 (0.327, 0.871)	0.012^*^	68.2%	0.024	
**CD57**								
OS	27	4,399	R	0.484 (0.380, 0.616)	<0.001^*^	77.4%	<0.001	0.707
OS from M	10	1,129	R	0.525 (0.346, 0.797)	0.003^*^	81.7%	<0.001	0.721
DFS	6	1,004	F	0.543 (0.404, 0.729)	<0.001^*^	0.0%	0.444	—
PFS	2	147	F	0.454 (0.204, 1.009)	0.053	0.0%	0.841	—
RFS	3	2,156	R	0.653 (0.374, 1.140)	0.134	65.0%	0.057	—
**NKp30**								
OS	1	61	—	0.34 (0.14, 0.80)	0.014^*^	—	—	—
**NKp46**								
OS	5	953	F	0.622 (0.470, 0.821)	0.001^*^	35.1%	0.187	0.462
OS from M	2	286	F	0.559 (0.385, 0.812)	0.002^*^	48.6%	0.163	—
DFS	2	389	F	0.932 (0.626, 1.386)	0.727	49.1%	0.161	—
PFS	1	53	—	0.20 (0.047, 0.856)	0.03^*^	—	—	—
RFS	2	181	F	0.591 (0.388, 0.900)	0.014^*^	25.2%	0.248	—

M, multivariate analysis; R, random-model; F, fixed-model;

* *p < 0.05*.

Furthermore, we assessed the prognostic significance of CD56 in certain types of tumors. The results showed that a high level of CD56 predicted better OS in head and neck squamous cell carcinoma (HNSCC) (HR = 0.356, 95%CI: 0.237–0.533, *p* < 0.001) (Supplementary Figure 1A), but not in colorectal cancer (CRC) (HR = 0.574, 95%CI: 0.328–1.004, *p* = 0.052) (Supplementary Figure 1B), glioma (HR = 0.933, 95%CI: 0.050–17.523, *p* = 0.963) (Supplementary Figure 1C), or HCC (HR = 0.620, 95%CI: 0.124–3.109, *p* = 0.561) (Supplementary Figure 1D; Table 3).

**Table 3:** Pooled HR and heterogeneity of the meta-analysis of OS in patients with certain types of solid tumors.

**Factor**	**No. of cohorts**	**Model**	**Pooled HR (95%CI)**	***P***	**Heterogeneity**	
					***I*^2^**	***P***
**CD56**						
OS for CRC	4	R	0.574 (0.328, 1.004)	0.052	62.9%	0.044
OS for glioma	2	R	0.933 (0.050, 17.523)	0.963	88.7%	0.003
OS for HCC	2	R	0.620 (0.124, 3.109)	0.561	79.2%	0.028
OS for HNSCC	4	F	0.356 (0.237, 0.533)	<0.001^*^	31.4%	0.224
**CD57**						
OS for CRC	5	F	0.529 (0.376, 0.746)	<0.001^*^	34.6%	0.191
OS for ESCC	4	F	0.577 (0.426, 0.782)	<0.001^*^	34.3%	0.206
OS for GC	3	F	0.583 (0.395, 0.861)	0.007^*^	0.0%	0.405
OS for HCC	2	F	0.528 (0.367, 0.760)	0.001^*^	40.8%	0.194
OS for HNSCC	5	R	0.326 (0.136, 0.785)	0.012^*^	76.9%	0.002
OS for NSCLC	2	F	0.301 (0.172, 0.528)	<0.001^*^	0.0%	0.383
OS for RCC	2	R	0.713 (0.361, 1.406)	0.329	84.7%	0.010

R, random-model; F, fixed-model;

* *p < 0.05*.

The impact of CD56 on the prognosis of solid malignancies was investigated in 2 studies comprising 245 cases for DFS and 4 studies comprising 379 cases for PFS. A fixed-effect model was appropriate for the analysis of DFS (Cochrane Q, *p* = 0.310; *I*^2^ = 2.9%), while random effect model was used for PFS due to obvious heterogeneity (Cochrane Q, *p* = 0.024; *I*^2^ = 68.2%). The results displayed that CD56 was significantly correlated with favorable DFS (HR = 0.274, 95%CI: 0.111–0.679, *p* = 0.005) (Supplementary Figure 1E) and favorable PFS (HR = 0.533, 95%CI: 0.327–0.871, *p* = 0.012) (Supplementary Figure 1F) in patients with solid tumors (Table 2).

### Prognostic Value of NK Cell Marker CD57 in Patients With Solid Tumors

**Figure 3:**
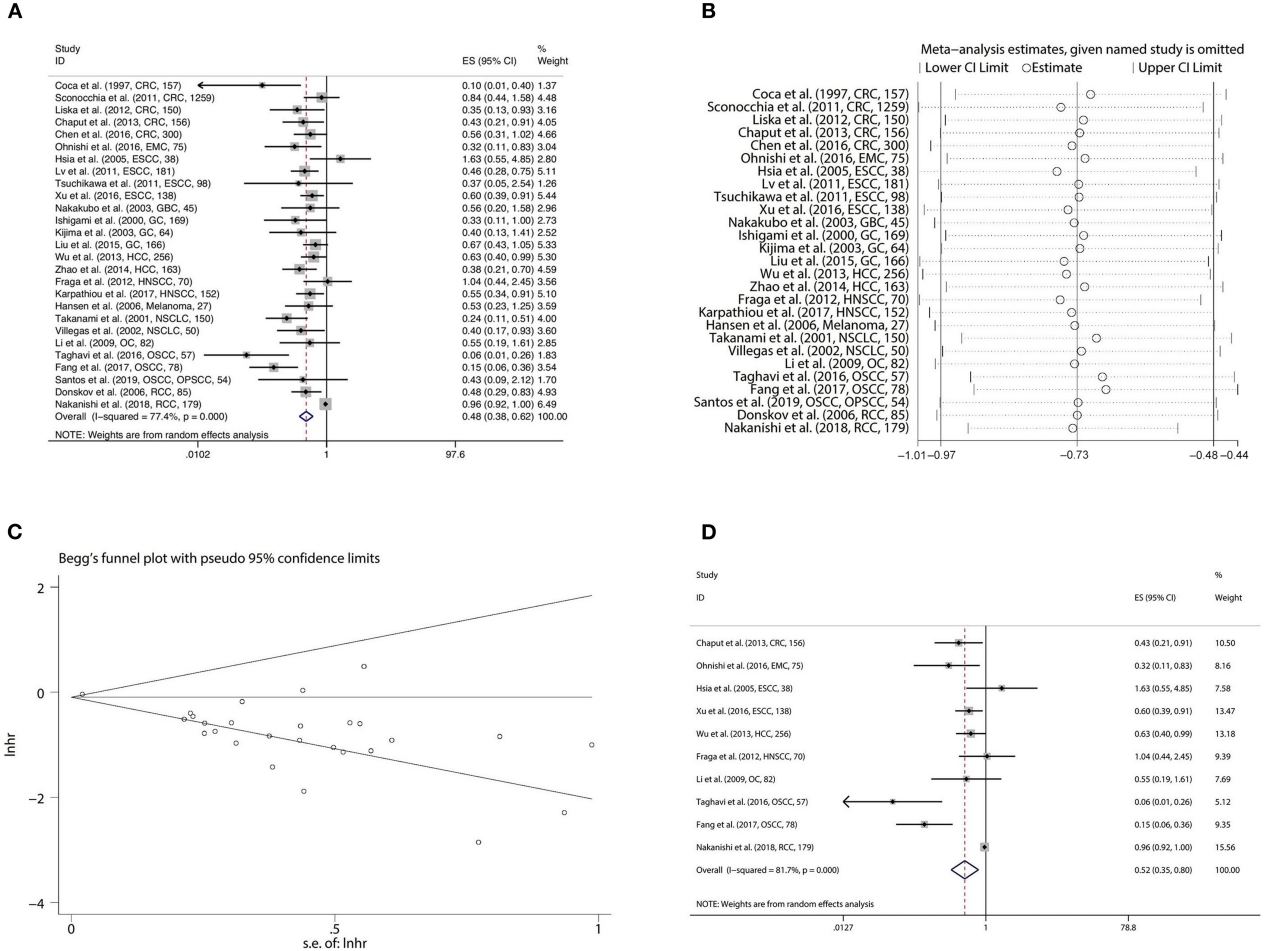
Forest plot **(A)**, sensitivity analysis plot **(B)**, and Begg's test **(C)** of the meta-analysis of OS for solid tumor patients divided by the level of CD57. Forest plot **(D)** of the meta-analysis of OS for solid tumor patients divided by the level of CD57, from Cox multivariate analysis.

To estimate the prognostic significance of CD57 in OS, we pooled the data from 27 studies involving 4,399 patients. A random effect model was more appropriate in the analysis of OS due to the obvious inter-study heterogeneity (Cochrane Q, *p* < 0.001; *I*^2^ = 77.4%). Our results revealed CD57 to be significantly correlated with favorable OS in patients with solid tumors (HR = 0.484, 95%CI: 0.380–0.616, *p* < 0.001) (Figure 3A; Table 2). The original statistical significance was not altered after omitting any cohorts in the sensitivity analysis (Figure 3B), and publication bias was not detected by Begg's test (*p* = 0.707) (Figure 3C). In addition, pooled HR of 10 pieces of research conducting Cox multivariate analysis showed that a high level of CD57 in solid tumors could independently predict better OS (HR = 0.525, 95%CI: 0.346–0.797, *p* = 0.003) (Figure 3D; Table 2).

The prognostic significance of CD57 in certain types of tumors was further assessed. CD57 predicted improved OS in colorectal cancer (CRC) (HR = 0.529, 95%CI: 0.376–0.746, *p* < 0.001) (Supplementary Figure 2A), esophageal squamous cell carcinoma (ESCC) (HR = 0.577, 95%CI: 0.426–0.782, *p* < 0.001) (Supplementary Figure 2B), gastric cancer (GC) (HR = 0.583, 95%CI: 0.395–0.861, *p* = 0.007) (Supplementary Figure 2C), HCC (HR = 0.528, 95%CI: 0.367–0.760, *p* = 0.001) (Supplementary Figure 2D), HNSCC (HR = 0.326, 95%CI: 0.136–0.785, *p* = 0.012) (Supplementary Figure 2E), and non-small cell lung cancer (NSCLC) (HR = 0.301, 95%CI: 0.172–0.528, *p* < 0.001) (Supplementary Figure 2F), but not in renal cell carcinoma (RCC) (HR = 0.713, 95%CI: 0.361–1.406, *p* = 0.329) (Supplementary Figure 2G; Table 3).

Furthermore, the impact of CD57 on the prognosis of solid malignancies was investigated in 6 studies comprising 1,004 cases for DFS, 2 studies comprising 147 cases for PFS, and 3 studies comprising 2,156 cases for RFS. A fixed-effect model was preferred in the analyses of DFS (Cochrane Q, *p* = 0.444; *I*^2^ = 0.0%) and PFS (Cochrane Q, *p* = 0.841; *I*^2^ = 0.0%), while random effect model was used for RFS owing to obvious heterogeneity (Cochrane Q, *p* = 0.057; *I*^2^ = 65.0%). The results displayed that CD57 was significantly associated with better DFS in patients with solid malignancies (HR = 0.543, 95%CI: 0.404–0.729, *p* < 0.001) (Supplementary Figure 2H). However, the prognostic significance of CD57 was not detected in the analysis of PFS (HR = 0.454, 95%CI: 0.204–1.009, *p* = 0.053) (Supplementary Figure 2I) or RFS (HR = 0.653, 95%CI: 0.374–1.140, *p* = 0.134) (Supplementary Figure 2J; Table 2).

### Prognostic Value of NK Markers NKp30 and NKp46 in Patients With Solid Tumors

Only one piece of original research (77) conducting RT-PCR of NKp30 was included in the current study, which showed that a high level of NKp30 could predict better OS of HCC patients (HR = 0.34, 95%CI: 0.14–0.80, *p* = 0.014) (Table 2). Thus, the prognostic value of NKp30 is inconclusive in the current meta-analysis due to limited eligible studies.

**Figure 4:**
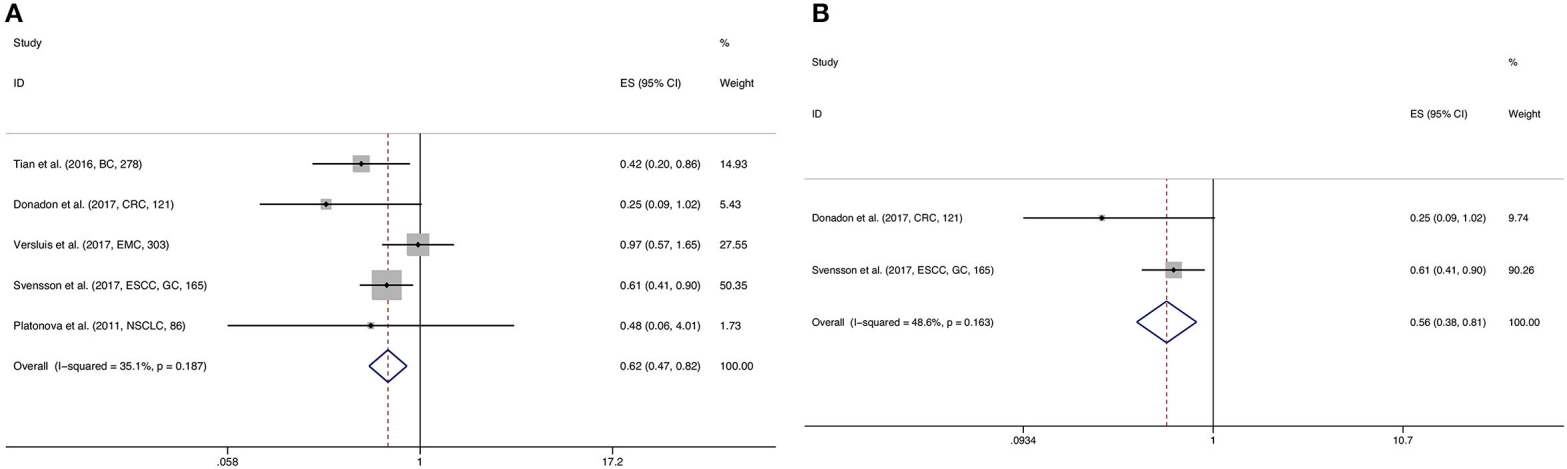
Forest plot **(A)** of the meta-analysis of OS for solid tumor patients divided by the level of NKp46. Forest plot **(B)** of the meta-analysis of OS for solid tumor patients divided by the level of NKp46, from Cox multivariate analysis.

The prognostic value of NKp46 was explored in 7 observation studies, including 5 for OS, 2 for DFS, 1 for PFS, and 2 for RFS. NKp46 was significantly correlated with favorable OS (HR = 0.622, 95%CI: 0.470–0.821, *p* = 0.001) (Figure 4A) and RFS (HR = 0.591, 95%CI: 0.388–0.900, *p* = 0.014). However, statistical significance of NKp46 in DFS was not detected by the present meta-analysis (HR = 0.932, 95%CI: 0.626–1.386, *p* = 0.727) (Table 2). In addition, pooled HR of two Cox multivariate analyses demonstrated that NKp46 was an independent prognostic predictor of better OS (HR = 0.559, 95%CI: 0.385–0.812, *p* = 0.002) (Figure 4B; Table 2).

## Discussion

TINKs play a significant role in tumor defense, which might be related to tumor prognosis. However, inconsistent results were reported by previous clinical studies and some studies suggested that TINKs are not capable of efficient tumor lysis (85, 86). The present meta-analysis comprehensively analyzed the prognostic significance of NK cell markers in solid tumors. We analyzed the results of 56 independent original studies and detected that high levels of NK cell markers CD56, CD57, NKp30, or NKp46 were all correlated with better prognosis of solid tumor patients.

NK cells were defined as CD3^−^CD56^+^ lymphocytes. Based on the density of CD56, NK cells are divided into CD56^bright^ and CD56^dim^ subsets. CD56 could drive the maturation of NK cells and act as a reliable marker for TINKs (87). Normally, more than 90% of PBNKs are CD16^+^CD56^dim^ NK cells, which are a more matured and more cytotoxic subset, while CD56^bright^ NK cells are a less matured subset that comprise 5–10% of PBNKs (11, 88). CD56^bright^ NK cells were considered as strong “cytokine producers” that secrete anti-tumor cytokines such as IFN-γ in response to IL-2, IL-12, and IL-18 (24). Carrega et al. also indicated that NK cells infiltrating NSCLC mainly exerted cytokine producing effects rather than direct tumor cell killing effects (89). Correspondingly, the current meta-analysis indicated that a high level of CD56 could predict improved OS, DFS, and PFS in solid tumor patients. In addition, our results indicated that CD56 could serve as an independent predictor of improved OS. Nevertheless, discrepant results were reached in the analysis of certain tumor types, which might be attributed to the limited number of eligible studies.

CD57 is another crucial surface marker of NK cells, which represents the terminal maturation of NK cells (22, 90). CD57^+^ NK cells are featured with less proliferation and less IFN-γ production, but with higher cytotoxicity to tumor cells in comparison with CD57^−^ NK cells (25). Previous studies also reported that CD57^+^ NK cells could reacquire the IFN-γ producing potential when crosslinked with CD16 (22, 91). Therefore, appropriate proportion of CD57^+^ and CD57^−^ NK cells could have synergistic anti-tumor effects, and therefore benefit the survival of tumor-bearing patients (92, 93). After stimulation by tumor-associated antigens, CD57^+^ NK cells are accumulated in various solid tumor tissues (48–50, 94). Thus, a great deal of research attempted to clarify the clinical role of a high level of CD57^+^ TINKs, which might be a signal for effective tumor defense. We analyzed the data from 31 included studies and the results suggested that a high level of CD57 was correlated with improved OS and DFS in patients with solid tumors in the current meta-analysis. Moreover, high level of CD57 predicted improved OS in CRC, ESCC, GC, HCC, HNSCC, and NSCLC in the analysis of certain types of tumors.

In recent decades, three natural cytotoxicity receptor (NCR) members of NK cells have been identified, including NKp30 (NCR3), NKp44 (NCR2), and NKp46 (NCR1). Six different splice variants of NKp30 (NKp30a-e) were expressed on the cell surface and each of them has a specific function (95). NKp30a and NKp30b were found to induce cytotoxicity and cytokine production respectively, whereas NKp30c showed an immunosuppressive activity in previous research (96). Only one study on NKp30 was included in this meta-analysis, which reported that a high level of NKp30 was associated with improved OS of HCC (77). Therefore, its prognostic value needs to be further explored. In terms of NKp46, Halfteck et al. found that tumor growth was enhanced in NKp46-deficient mice, which highlighted the vital role of NKp46^+^ NK cells in anti-tumor defense (97). Moreover, NKp46 could upregulate tumor necrosis factor-related apoptosis-inducing ligand (TRAIL), thus enhancing the anti-tumor effects of NK cells (98). Currently, our results indicated that a high level of NKp46 was significantly associated with favorable OS and RFS.

Our results imply some concerns regarding current clinical research. On the basis of TNM staging system, a more accurate prognosis prediction system according to the level of these markers could be built in the future, to direct the selection of therapeutic regimens. Further studies are highly encouraged to explore the regulatory mechanisms of the tumor-infiltrating processes of NK cells and the enhancement methods of the anti-tumor effects of TINKs (99). In addition, targeting the function state of NK cells might be a promising treatment strategy for solid tumors, such as IL-15-mediated CD56 activation (100). However, great care is needed when extrapolating the prognostic value into treatment potential.

Notably, discrepant results were achieved in the current study, which might decrease the evidence grade to some extent. The inconsistent results might be attributed to a limited number of included studies in a certain analysis. In addition, obvious inter-study heterogeneity existed in the analysis of the prognostic significance of CD56 and CD57 in OS. We speculated that the following factors might be the sources of heterogeneity: (1) Tumor types and clinical stages differed among included studies, (2) Detection and cut-off methodologies differed, and (3) Some included studies were featured with a small sample size or short follow-up period.

Finally, several limitations of this work should be underlined. Firstly, the detection and cut-off methodologies were discrepant among included studies, although IHC staining and scoring of the number of positive stained cells were conducted by most studies. Secondly, the data were not directly obtained from 18 included articles. The processes of calculating HRs and 95% CIs by survival curves might result in data inaccuracy to some extent. Thirdly, different pathological patterns, pathological grades, clinical stages, and therapeutic regimens among the included studies limited the credibility and applicability of the conclusions. Fourthly, although most included studies detected NK cell markers to identify the degree of NK cell tumor-infiltration, it should be stated that the prognostic value of NK cell markers might be attributed to the synthetic effects of multiple immune cells for the reason that other types of immune cells, such as NK-T cells, dendritic cells, and monocytes, partially shared some surface markers of NK cells (101). Fifthly, the potential publication bias in the current analysis should not be overlooked, which might be derived from the missing of non-English articles or unpublished results.

## Conclusions

Our results suggested that high levels of NK cell markers CD56, CD57, NKp30, and NKp46 were significantly correlated with favorable outcomes for solid tumor patients, providing theoretical evidence for further accurate prognosis prediction of solid tumors. However, to transfer the conclusions into a clinical application, a specific scoring system should be established and tested with prospective and high-quality researches.

## Data Availability Statement

The datasets analyzed for this study are available from the corresponding author (ZS).

## Author Contributions

ZS conceived of and designed the study. SZ and WL collected, extracted, and analyzed the data. BH, PW, XL, and SC performed the quality assessment and analyzed the data. SZ wrote the paper. All authors read and approved the manuscript.

## Conflict of Interest

The authors declare that the research was conducted in the absence of any commercial or financial relationships that could be construed as a potential conflict of interest.

## Abbreviations

TINK: tumor-infiltrating natural killer cell
HR: hazard ratio
CI: confidence interval
IFN: interferon
NCR: natural cytotoxicity receptor
IHC: immunohistochemical
FCM: flow cytometry
RT-PCR: real-time polymerase chain reaction
OS: overall survival
DFS: disease-free survival
MFS: metastasis-free survival
PFS: progression-free survival
RFS: recurrence-free survival
NOS: Newcastle-Ottawa Scale.
