# Supplementary material for: Prognostic Significance of Tumor-Infiltrating Natural Killer Cells in Solid Tumors: A Systematic Review and Meta-Analysis
Source: Front Immunol. 2020 Jul 2;11:1242. doi: 10.3389/fimmu.2020.01242 (PMC7343909; doi:10.3389/fimmu.2020.01242)
Supplement: Supplementary file 1 [file Table_1.docx]

**Supplementary Table 1. Characteristics of included studies**

| **Surface marker** | **Author** | **Year** | **Region** | **Tumor type** | **Clinical stage** | **Detection method** | **Sample size** | **Data extraction^a^** | **Endpoint** | **Follow-up period** | **Nos score** | **Citation** |
| --- | --- | --- | --- | --- | --- | --- | --- | --- | --- | --- | --- | --- |
| CD56 | van Herpen et al. | 2005 | the Netherlands | HNSCC | NS | IHC | 27 | 2 | OS | 5.5y | 6 | (32) |
|  | Al-Shibli et al. | 2009 | Norway | NSCLC | I-IIIa | IHC | 332 | 1 | OS | Md: 96m; R: 10-179m | 6 | (33) |
|  | Maréchal et al. | 2010 | Belgium | CRC | NS | IHC | Cohort 1: 33 Cohort 2: 35 | 1 | OS, PFS | 20m | 6 | (34) |
|  | Sconocchia et al. | 2011 | NS | CRC | NS | IHC | 1203 | 1 | OS | 60m | 6 | (35) |
|  | Eckl et al. | 2012 | Germany | RCC | I-IV | FCM | 30 | 2 | OS | Md: 45m; R: 3-139m | 6 | (36) |
|  | Chew et al. | 2012 | Singapore | HCC | I-IV | IHC | 36 | 1 | OS | 9y | 6 | (37) |
|  | Lin et al. | 2013 | China | HCC | I-III | IHC | 132 | 1 | OS, DFS | 72m | 7 | (38) |
|  | Rathore et al. | 2014 | India | BC | NS | IHC | 175 | 1 | OS | 4y | 7 | (30) |
|  | Sznurkowski et al. | 2014 | Poland | VSCC | FIGO I-IV | IHC | 75 | 2 | OS | Md: 51m; R: 6-136m | 6 | (39) |
|  | Amoueian et al. | 2015 | Iran | GC | I-III | IHC | 50 | 2 | OS | 80m | 8 | (27) |
|  | Wagner et al. | 2016 | Germany | OSCC | I-IV | IHC | 140 | 1 | OS | 5y | 6 | (40) |
|  | Alderdice et al. | 2017 | the U.K. | CRC | NS | IHC | 149 | 1 | OS | R: 19-99m | 7 | (41) |
|  | Lu et al. | 2018 | China | NPC | I-IV | IHC | 197 | 1 | OS, PFS | Md: 51m; M: 50m | 7 | (42) |
|  | Stangl et al. | 2018 | Germany | HNSCC | NS | IHC | 114 | 1 | OS, PFS, MFS | 24m | 6 | (43) |
|  | Inaguma et al. | 2019 | NS | MPM | NS | IHC | 63 | 2 | OS | 5y | 6 | (44) |
|  | Muntasell et al. | 2019 | Spain | BC | NS | IHC | Cohort 1: 42  Cohort 2: 71 | 1 | DFS | Cohort 1, Md: 31m; IQR: 22‐38m; Cohort 2, Md: 49m; IQR:24‐71m | 6 | (45) |
|  | Ren et al. | 2019 | China | Glioma | NS | RT-PCR | 61 | 2 | OS | 45m | 6 | (46) |
|  | Wu et al. | 2019 | China | Glioma | NS | IHC | 30 | 2 | OS | 60m | 6 | (47) |
| CD57 | Coca et al. | 1997 | Spain | CRC | I–III | IHC | 157 | 1 | OS, DFS | 5y | 8 | (48) |
|  | Ishigami et al. | 2000 | Japan | GC | NC | IHC | 169 | 2 | OS | M: 63m; R: 13-122m | 7 | (49) |
|  | Takanami et al. | 2001 | Japan | NSCLC | I–IIIa | IHC | 150 | 2 | OS | R: 60-120m | 8 | (50) |
|  | Villegas et al. | 2002 | Spain | NSCLC | I–IIIa | IHC | 50 | 1 | OS | M: 56m; R: 6-149m | 6 | (51) |
|  | Kijima et al. | 2003 | Japan | GC | I–IV | IHC | 64 | 1 | OS | Md: 30m; R: 1-176m | 6 | (52) |
|  | Nakakubo et al. | 2003 | Japan | GBC | I–IV | IHC | 45 | 1 | OS | 5y | 6 | (53) |
|  | Menon et al. | 2004 | the Netherlands | CRC | II–III | IHC | 87 | 1 | DFS | Md: 73m; R: 1-223m | 6 | (54) |
|  | Hsia et al. | 2005 | China | ESCC | I–IV | IHC | 38 | 2 | OS | 100m | 6 | (29) |
|  | Donskov et al. | 2006 | Denmark | RCC | IV | IHC | 85 | 1 | OS | Md: 57m; R: 32-73m | 7 | (55) |
|  | Hansen et al. | 2006 | Denmark | Melanoma | IV | IHC | 27 | 1 | OS | Md: 9m; R: 1-35m | 6 | (56) |
|  | Ino et al. | 2008 | Japan | EMC | FIGO I-IV | IHC | 65 | 1 | PFS | Md: 72m; R: 5-148m | 6 | (57) |
|  | Li et al. | 2009 | Japan | OC | FIGO I-IV | IHC | 82 | 1 | OS, PFS | M: 68m; R: 1-154m | 6 | (58) |
|  | Lv et al. | 2011 | China | ESCC | I–IV | IHC | 181 | 2 | OS | 80m | 7 | (59) |
|  | Sconocchia et al. | 2011 | NS | CRC | NS | IHC | 1259 | 1 | OS | 60m | 6 | (35) |
|  | Tsuchikawa et al. | 2011 | Japan | ESCC | I–IV | IHC | 98 | 2 | OS | 8y | 7 | (60) |
|  | Fraga et al. | 2012 | Brazil | HNSCC | I–IV | IHC | 70 | 1 | OS | 35m | 6 | (61) |
|  | Liska et al. | 2012 | Czech Republic | CRC | NS | IHC | 150 | 2 | OS, DFS | 4.7y | 6 | (62) |
|  | Chaput et al. | 2013 | France | CRC | II–III | IHC | 156 | 1 | OS, RFS | Md: 10y; IQR: 6.8-13.3y | 8 | (63) |
|  | Wu et al. | 2013 | China | HCC | I–IV | IHC | 256 | 1 | OS, DFS | 80m | 7 | (64) |
|  | Wangerin et al. | 2014 | Germany | PC | NS | IHC | 1916 | 1 | RFS | M: 34m; R: 1-144m | 7 | (65) |
|  | Zhao et al. | 2014 | China | HCC | NS | IHC | 163 | 2 | OS | 80m | 8 | (66) |
|  | Hernandez-Prieto et al. | 2015 | Spain | NSCLC | I–II | IHC | 84 | 1 | RFS | 115m | 7 | (67) |
|  | Liu et al. | 2015 | China | GC | I–IV | IHC | 166 | 1 | OS | Md: 66m | 8 | (68) |
|  | Chen et al. | 2016 | China | CRC | I–IV | IHC | 300 | 1 | OS, DFS | M: 63m | 6 | (69) |
|  | Ohnishi et al | 2016 | Japan | EMC | I–IV | IHC | 75 | 1 | OS | 120m | 7 | (70) |
|  | Taghavi et al. | 2016 | Iran | OSCC | NS | IHC | 57 | 1 | OS | M: 29m; R: 10-85m | 7 | (71) |
|  | Xu et al. | 2016 | China | ESCC | II–III | IHC | 138 | 1 | OS | 10y | 7 | (72) |
|  | Fang et al. | 2017 | China | OSCC | I–IV | IHC | 78 | 1 | OS | Md: 48m; R: 29-93m | 7 | (73) |
|  | Karpathiou et al. | 2017 | France | HNSCC | I–IV | IHC | 152 | 2 | OS | Md: 24m; R: 3-84m | 6 | (74) |
|  | Nakanishi et al. | 2018 | Japan | RCC | NS | IHC | 179 | 1 | OS | Md: 39m; IQR: 19-62m | 7 | (75) |
|  | Santos et al. | 2019 | Brazil | OSCC, OPSCC | II–IV | IHC | 54 | 2 | OS, DFS | 80m | 7 | (76) |
| NKp30 (NCR3) | Chew et al. | 2010 | Singapore | HCC | I-IIIa | RT-PCR | 61 | 1 | OS | Md: 2.56y; R: 0.02-9.11y | 6 | (77) |
| NKp46 (NCR1) | Platonova et al. | 2011 | France | NSCLC | I-III | IHC | 86 | 2 | OS, DFS | 35m | 6 | (78) |
|  | Ascierto et al. | 2013 | the U.S. | BC | I-III | RT-PCR | 16 | 1 | RFS | 80m | 6 | (79) |
|  | Rusakiewicz et al. | 2013 | France, Spain | GIST | NS | IHC | 53 | 1 | PFS | Md: 51m | 6 | (80) |
|  | Tian et al. | 2016 | China | BC | I-III | IHC | 278 | 2 | OS | Md: 76 m; R: 4-116m | 7 | (81) |
|  | Donadon et al. | 2017 | Italy | CRC | IV | IHC | 121 | 1 | OS | Md: 35m; R: 2-117m | 6 | (82) |
|  | Svensson et al. | 2017 | Sweden | ESCC, GC | NS | IHC | 165 | 1 | OS, RFS | 9y | 7 | (83) |
|  | Versluis et al. | 2017 | the Netherlands | EMC | FIGO I-IV | IHC | 303 | 1 | OS, DFS | Md: 6.2y; IQR: 2.4-10.5y | 8 | (84) |

ESCC, esophageal squamous cell carcinoma; GC, gastric cancer; CRC, colorectal cancer; HCC, hepatocellular carcinoma; ICC, intrahepatic cholangiocarcinoma; GBC, gallbladder cancer; GIST, gastrointestinal stromal tumor; OSCC, oral squamous cell carcinoma; OPSCC, oropharyngeal squamous cell carcinoma; NPC, nasopharyngeal carcinoma; HNSCC, head and neck squamous cell carcinoma; NSCLC, non-small cell lung cancer; RCC, renal cell carcinoma; PC: prostatic cancer; MPM: malignant pleural mesothelioma; BC, breast cancer, OC, ovarian cancer; VSCC, vulvar squamous cell carcinoma; EMC, endometrial cancer; IHC: immunohistochemical staining; FCM: flow cytometry; RT-PCR: real-time polymerase chain reaction; FIGO, International Federation of Gynecology and Obstetrics; OS, overall survival; DFS, disease-free survival; MFS, metastasis-free survival; PFS, progression-free survival; RFS, recurrence-free survival; NOS, Newcastle-Ottawa Scale; NS, not stated; M, mean; Md, median; R, range; IQR, interquartile range

a: 1 represented that HRs and 95%CIs were directly obtained from the publications; 2 represented that HRs and 95%CIs were derived from Kaplan-Meier curves.
