# Supplementary material for: Prognostic Significance of Tumor-Infiltrating Natural Killer Cells in Solid Tumors: A Systematic Review and Meta-Analysis
Source: Front Immunol. 2020 Jul 2;11:1242. doi: 10.3389/fimmu.2020.01242 (PMC7343909; doi:10.3389/fimmu.2020.01242)
Supplement: Supplementary file 2 [file Table_2.DOCX]

**Supplementary Table 2** **NOS scores of included studies**

| **Author** | **Year** | **Selection** | | | | **Comparability** | | **Outcome** | | | **Nos score** |
| --- | --- | --- | --- | --- | --- | --- | --- | --- | --- | --- | --- |
|  |  | **Item 1^a^** | **Item 2^b^** | **Item 3^c^** | **Item 4^d^** | **Item 5^e^** | **Item 6^f^** | **Item 7^g^** | **Item 8^h^** | **Item 9^i^** |  |
| van Herpen et al. | 2005 | 0 | 0 | 1 | 1 | 1 | 1 | 1 | 1 | 0 | 6 |
| Al-Shibli et al. | 2009 | 0 | 1 | 1 | 1 | 1 | 0 | 1 | 1 | 0 | 6 |
| Maréchal et al. | 2010 | 1 | 1 | 1 | 1 | 0 | 1 | 1 | 0 | 0 | 6 |
| Sconocchia et al. | 2011 | 1 | 1 | 1 | 1 | 0 | 0 | 1 | 1 | 0 | 6 |
| Eckl et al. | 2012 | 0 | 1 | 0 | 1 | 1 | 0 | 1 | 1 | 1 | 6 |
| Chew et al. | 2012 | 1 | 1 | 0 | 1 | 0 | 1 | 1 | 1 | 0 | 6 |
| Lin et al. | 2013 | 1 | 1 | 1 | 1 | 1 | 0 | 1 | 1 | 0 | 7 |
| Rathore et al. | 2014 | 1 | 1 | 1 | 1 | 1 | 1 | 1 | 0 | 0 | 7 |
| Sznurkowski et al. | 2014 | 1 | 0 | 1 | 1 | 0 | 1 | 1 | 1 | 0 | 6 |
| Amoueian et al. | 2015 | 1 | 1 | 1 | 1 | 1 | 0 | 1 | 1 | 1 | 8 |
| Wagner et al. | 2016 | 1 | 1 | 1 | 1 | 0 | 0 | 1 | 1 | 0 | 6 |
| Alderdice et al. | 2017 | 1 | 1 | 1 | 1 | 1 | 0 | 1 | 1 | 0 | 7 |
| Lu et al. | 2018 | 1 | 1 | 1 | 1 | 1 | 0 | 1 | 1 | 0 | 7 |
| Stangl et al. | 2018 | 1 | 1 | 1 | 1 | 1 | 0 | 1 | 0 | 0 | 6 |
| Inaguma et al. | 2019 | 1 | 1 | 1 | 1 | 0 | 0 | 1 | 1 | 0 | 6 |
| Muntasell et al. | 2019 | 1 | 1 | 1 | 1 | 1 | 0 | 1 | 0 | 0 | 6 |
| Ren et al. | 2019 | 1 | 1 | 1 | 1 | 1 | 0 | 1 | 0 | 0 | 6 |
| Wu et al. | 2019 | 1 | 1 | 1 | 1 | 0 | 0 | 1 | 1 | 0 | 6 |
| Coca et al. | 1997 | 1 | 1 | 1 | 1 | 1 | 0 | 1 | 1 | 1 | 8 |
| Ishigami et al. | 2000 | 1 | 1 | 1 | 1 | 1 | 0 | 1 | 1 | 0 | 7 |
| Takanami et al. | 2001 | 1 | 1 | 0 | 1 | 1 | 1 | 1 | 1 | 1 | 8 |
| Villegas et al. | 2002 | 1 | 1 | 0 | 1 | 1 | 0 | 1 | 1 | 0 | 6 |
| Kijima et al. | 2003 | 0 | 1 | 1 | 1 | 1 | 0 | 1 | 1 | 0 | 6 |
| Nakakubo et al. | 2003 | 0 | 1 | 1 | 1 | 1 | 0 | 1 | 1 | 0 | 6 |
| Menon et al. | 2004 | 1 | 1 | 1 | 1 | 0 | 0 | 1 | 1 | 0 | 6 |
| Hsia et al. | 2005 | 0 | 1 | 1 | 1 | 1 | 0 | 1 | 1 | 0 | 6 |
| Donskov et al. | 2006 | 1 | 1 | 1 | 1 | 1 | 0 | 1 | 1 | 0 | 7 |
| Hansen et al. | 2006 | 0 | 0 | 1 | 1 | 1 | 0 | 1 | 1 | 1 | 6 |
| Ino et al. | 2008 | 1 | 1 | 1 | 1 | 0 | 0 | 1 | 1 | 0 | 6 |
| Li et al. | 2009 | 1 | 1 | 1 | 1 | 0 | 0 | 1 | 1 | 0 | 6 |
| Lv et al. | 2011 | 1 | 1 | 1 | 1 | 1 | 0 | 1 | 1 | 0 | 7 |
| Tsuchikawa et al. | 2011 | 1 | 1 | 1 | 1 | 1 | 0 | 1 | 1 | 0 | 7 |
| Fraga et al. | 2012 | 1 | 1 | 1 | 1 | 1 | 0 | 1 | 0 | 0 | 6 |
| Liska et al. | 2012 | 1 | 1 | 1 | 1 | 1 | 0 | 1 | 0 | 0 | 6 |
| Chaput et al. | 2013 | 1 | 1 | 1 | 1 | 1 | 0 | 1 | 1 | 1 | 8 |
| Wu et al. | 2013 | 1 | 1 | 1 | 1 | 1 | 0 | 1 | 1 | 0 | 7 |
| Wangerin et al. | 2014 | 1 | 1 | 1 | 1 | 0 | 0 | 1 | 1 | 1 | 7 |
| Zhao et al. | 2014 | 1 | 1 | 1 | 1 | 1 | 0 | 1 | 1 | 1 | 8 |
| Hernandez-Prieto et al. | 2015 | 1 | 1 | 1 | 1 | 1 | 0 | 1 | 1 | 0 | 7 |
| Liu et al. | 2015 | 1 | 1 | 1 | 1 | 0 | 1 | 1 | 1 | 1 | 8 |
| Chen et al. | 2016 | 1 | 1 | 0 | 1 | 1 | 0 | 1 | 1 | 0 | 6 |
| Ohnishi et al | 2016 | 1 | 1 | 1 | 1 | 1 | 0 | 1 | 1 | 0 | 7 |
| Taghavi et al. | 2016 | 1 | 1 | 1 | 1 | 0 | 0 | 1 | 1 | 1 | 7 |
| Xu et al. | 2016 | 1 | 1 | 1 | 1 | 1 | 0 | 1 | 1 | 0 | 7 |
| Fang et al. | 2017 | 1 | 1 | 1 | 1 | 0 | 1 | 1 | 1 | 0 | 7 |
| Karpathiou et al. | 2017 | 1 | 1 | 1 | 1 | 0 | 0 | 1 | 1 | 0 | 6 |
| Nakanishi et al. | 2018 | 1 | 1 | 1 | 1 | 1 | 0 | 1 | 1 | 0 | 7 |
| Santos et al. | 2019 | 1 | 1 | 1 | 1 | 1 | 0 | 1 | 1 | 0 | 7 |
| Chew et al. | 2010 | 1 | 1 | 1 | 1 | 0 | 0 | 1 | 1 | 0 | 6 |
| Platonova et al. | 2011 | 1 | 1 | 1 | 1 | 1 | 0 | 1 | 0 | 0 | 6 |
| Ascierto et al. | 2013 | 0 | 0 | 1 | 1 | 1 | 0 | 1 | 1 | 1 | 6 |
| Rusakiewicz et al. | 2013 | 1 | 1 | 1 | 1 | 0 | 1 | 1 | 0 | 0 | 6 |
| Tian et al. | 2016 | 1 | 1 | 0 | 1 | 1 | 1 | 1 | 1 | 0 | 7 |
| Donadon et al. | 2017 | 0 | 1 | 1 | 1 | 1 | 0 | 1 | 1 | 0 | 6 |
| Svensson et al. | 2017 | 1 | 1 | 1 | 1 | 1 | 0 | 1 | 1 | 0 | 7 |
| Versluis et al. | 2017 | 1 | 1 | 1 | 1 | 1 | 1 | 1 | 1 | 0 | 8 |

^a^ Item 1: Representativeness of the exposed cohorts

^b^ Item 2: Selection of the non-exposed cohort

^c^ Item 3: Ascertainment of exposure

^d^ Item 4: Demonstration that outcome of interest was not present at start of study

^e^ Item 5: Study controls the most important factor

^f^ Item 6: Study controls any additional factor

^g^ Item 7: Assessment of outcome

^h^ Item 8: Was follow-up long enough for outcome to occur

^i^ Item 9: Adequacy of follow up of cohorts
