# Supplementary material for: Prognostic Significance of Tumor-Infiltrating Natural Killer Cells in Solid Tumors: A Systematic Review and Meta-Analysis
Source: Front Immunol. 2020 Jul 2;11:1242. doi: 10.3389/fimmu.2020.01242 (PMC7343909; doi:10.3389/fimmu.2020.01242)

**Supplementary Figure Legends**

**Supplementary Figure 1:** Forest plots of the meta-analysis of OS for patients with HNSCC (A), CRC (B), glioma (C), and HCC (D) divided by the level of CD56. Forest plots of the meta-analysis of DFS (E), and PFS (F) for solid tumor patients divided by the level of CD56.

**Supplementary Figure 2:** Forest plots of the meta-analysis of OS for patients with CRC (A), ESCC (B), GC (C), HCC (D), HNSCC (E), NSCLC (F), and RCC (G) divided by the level of CD57. Forest plots of the meta-analysis of DFS (H), PFS (I), and RFS (J) for solid tumor patients divided by the level of CD57.

**Supplementary Figure 1**
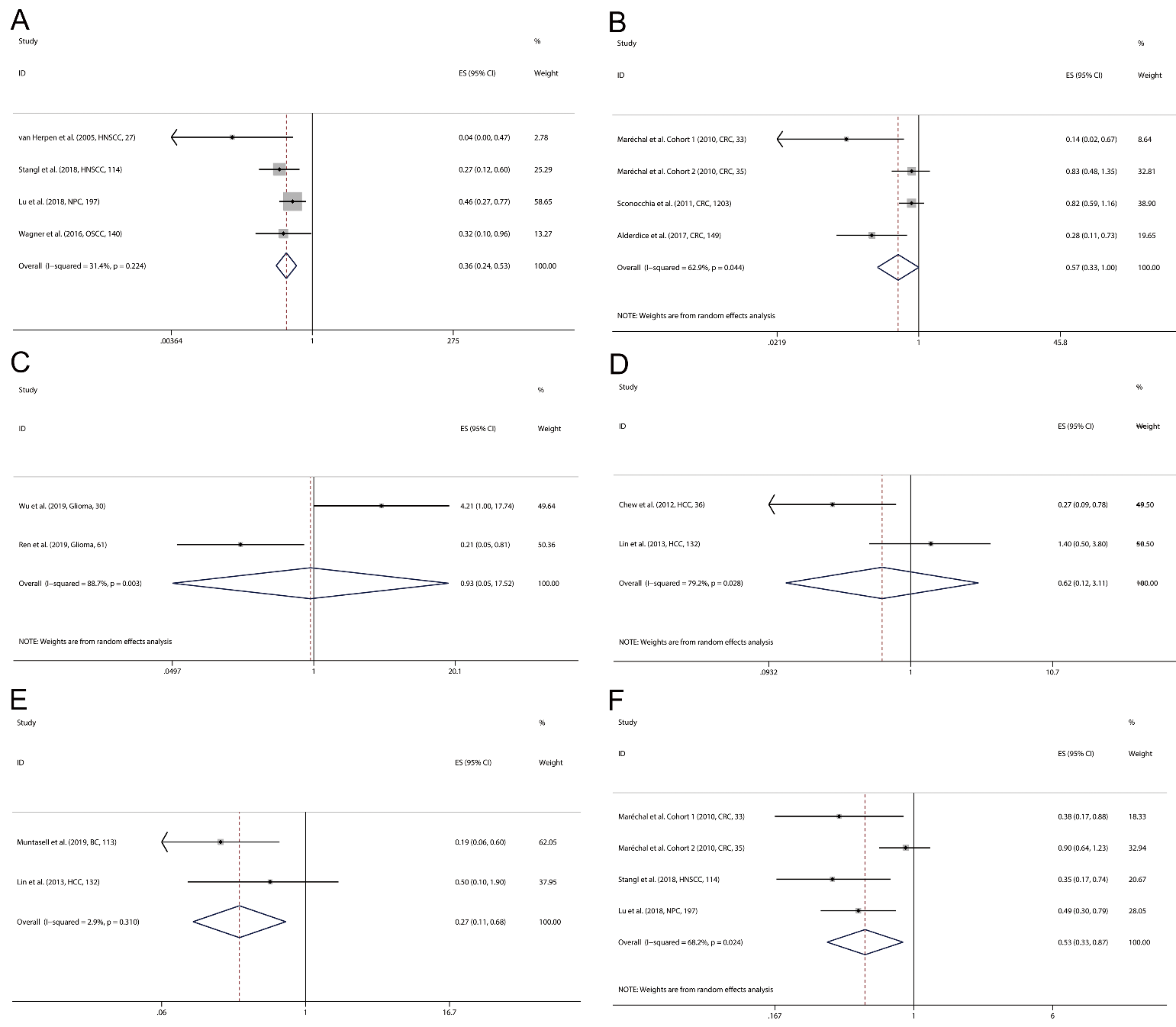


**Supplementary Figure 2**
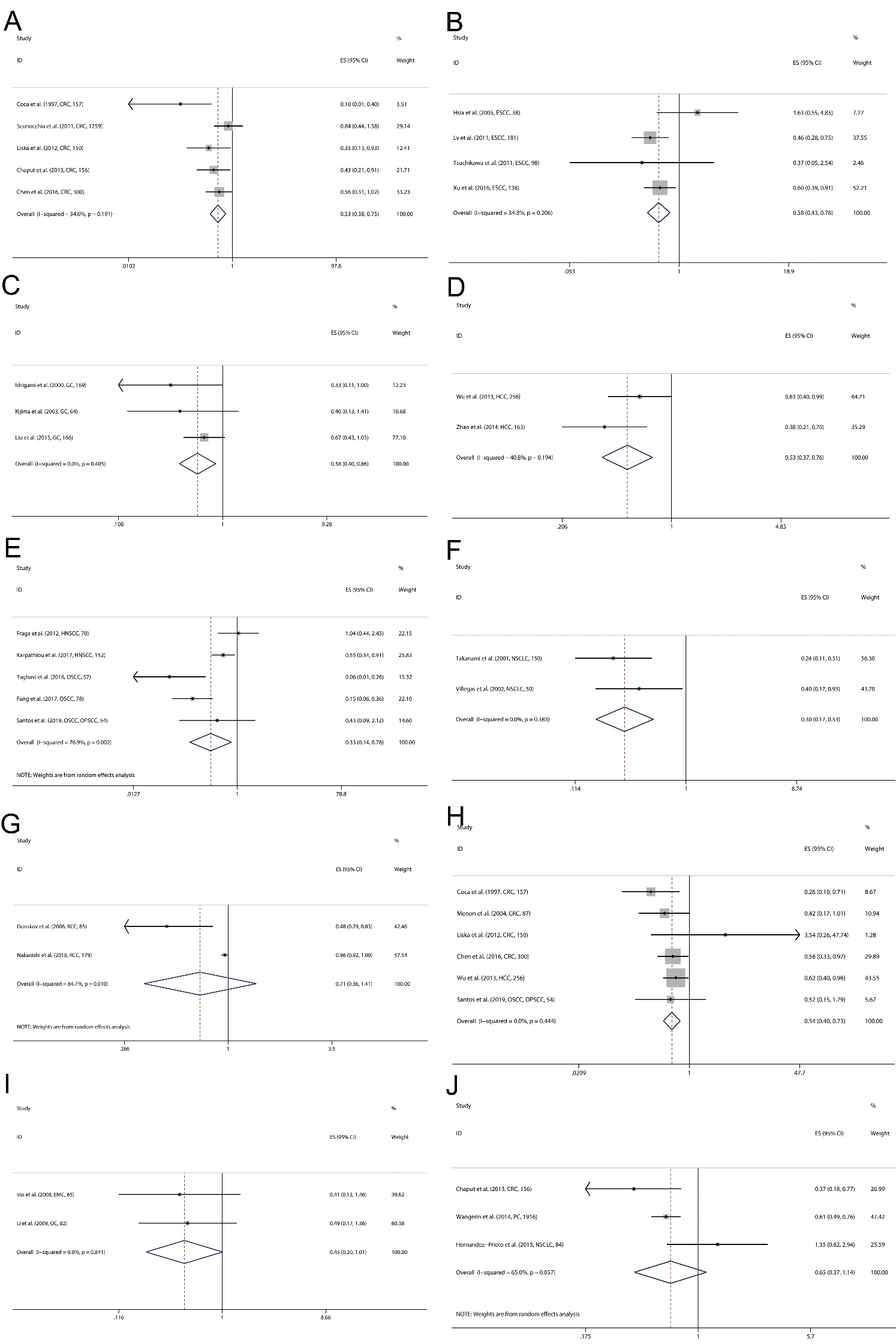

Supplement: Supplementary file 3 [file Data_Sheet_1.docx]
